# Green synthesis of carbon dot structures from *Rheum Ribes* and Schottky diode fabrication

**DOI:** 10.3762/bjnano.15.110

**Published:** 2024-11-07

**Authors:** Muhammed Taha Durmus, Ebru Bozkurt

**Affiliations:** 1 Department of Nanoscience and Nanoengineering, Graduate School of Natural and Applied Sciences, Atatürk University, 25240, Erzurum, Turkey; 2 Program of Occupational Health and Safety, Vocational College of Technical Sciences, Atatürk University, 25240 Erzurum, Turkey

**Keywords:** carbon dot (CD) structures, green synthesis, *Rheum Ribes* plant, Schottky diode

## Abstract

In this study, we aimed to synthesize new carbon dot structures (CDs) in a single step by using the plant *Rheum Ribes* for the first time and to contribute to the studies in the field of diode fabrication by using the new CDs. The CDs were obtained by hydrothermal synthesis, which is commonly used in the literature. TEM and zeta potential measurements were used to determine morphology and sizes of the CDs, and XRD, XPS, and FTIR and micro-Raman spectroscopy were used for structural characterization. Optical characterization of the CDs was done by absorption and steady-state fluorescence measurements. In the second part of the study, CDs were dripped onto silicon substrates, and a CDs thin film was formed by evaporation. A diode structure was obtained by evaporating gold with the shadow mask technique on the CDs film, and the current–voltage characteristics of this diode were examined. The synthesized CDs are spherical with an average size of 5.5 nm, have a negative surface charge and contain 73.3 atom % C, 24.0 atom % O, and 2.7 atom % N. The CDs exhibit fluorescence at approximately 394 nm. The layer thickness and bandgap energy of the prepared CDs film were calculated as 566 nm and 5.25 eV, respectively. The ideality factor and the measured barrier height (Φ_b_) of the CDs-based Schottky diode were calculated as 9.1 and 0.364 eV, respectively. The CDs were used as semiconductor material in a Schottky diode, and the diode exhibited rectification behavior. The results obtained from this study showed that CDs can be applied in the field of electronics, apart from sensor studies, which are common application areas.

## Introduction

One of the most current types of nanostructures are carbon dot structures (CDs). These structures have recently become a common field of study because of their properties including chemical stability, water solubility, and easy synthesis and functionalization. Carbon dots, were first discovered by Xu and his working group [1] while purifying single-walled carbon nanotubes in 2004. They are crystalline materials with dimensions between 1 and 10 nm, whose degree of carbonization can be changed, and exhibit many functional groups on the surface. CDs have attracted great attention because of their optical and chemical properties and have a wide range of uses in the fields of electrocatalysis, bioimaging, chemical sensors, biosensors, nanomedicine, biomolecule/drug release, light-emitting diodes, and photocatalysts. They also have promising applications in areas such as lasers and optoelectronic device applications [2–5].

CDs can be synthesized using many different materials. Natural materials have been used widely in recent studies [6–7]. The use of many plants, fruits, and different organic materials as natural carbon sources, abundant in nature, in the synthesis of CDs both reduces cost and prevents environmental pollution. These synthesis methods to obtain CDs from natural products are generally divided into top-down and bottom-up approaches, depending on the carbon source and the process used. In top-down syntheses, materials of desired size and structure are obtained from a bulk material. In bottom-up syntheses, larger nanostructures are obtained by using small nanoscale blocks. The hydrothermal synthesis method, which is a bottom-up method, is generally used in the synthesis of CDs. A very wide range of source materials, simple reaction equipment, and easy control of reaction conditions are the features that make this method superior to other methods [8–11].

*Rheum ribes*, a member of the sorrel family, is a plant species that grows among stones, rocks, and slopes at an altitude of 1000–4000 m, especially in the regions of eastern Turkey, Iran, Iraq, Lebanon, Afghanistan, and Pakistan. This 40–150 cm long perennial herbaceous plant with yellowish-white flowers grows from May to June. *Rheum ribes* is the only Rheum species growing in Turkey. Flavonoids, stilbenes, and anthraquinones in its structure are the main phenolic components that provide a potential antioxidant effect to this plant. The young shoots and leaf stems of *Rheum ribes* are used against diarrhea, stomach aches, and nausea, while other parts such as the root are used in the treatment of measles, pox, hemorrhoids, and as a bile expectorant [12–13].

One of the application areas of CDs are diodes, which are used to transmit and rectify a unidirectional electrical current. There are many types of diodes, such as Zener diodes, crystal diodes, and Schottky diodes, and each type has different features and is used for different purposes. Schottky diodes contain a metal–semiconductor junction. This structure differs from other diodes because of its high switching speed and low forward voltage [14].

In this study, CDs were synthesized in a single step by hydrothermal synthesis using *Rheum Ribes*, a natural material, for the first time, and diodes were fabricated using the new CDs. To our knowledge, studies on diodes with CDs obtained from natural materials are quite limited in the literature, which shows the importance of conducting this study. Hence, this study is to discuss the usability of CDs in a diode structure and does not focus on the development of device parameters in the literature. The results obtained from this study are important in terms of demonstrating the applicability of CDs in the field of electronics, apart from sensor studies, which are common application areas, and in terms of introducing new semiconductor materials to the field of electronics.

## Experimental

### Materials

*Rheum ribes* was purchased from the market, washed, and dried to synthesize CDs. Acetone, methanol, silicon, gold powder, and aluminum were purchased from Sigma-Aldrich, Merck, and Alfa Aesar. All studies were carried out at room temperature.

### Instruments

Various devices were used for the structural, morphological, and optical characterization of the CDs. FEI TALOS F200S TEM 200 kV, Malvern Zetasizer Nano ZSP, PANalytical X-ray diffractometer, Bruker VERTEX 70v, Specs‐Flex with a standard Al X‐ray source, WITech alpha 300R, VAKSIS PVD Handy, Zeiss Sigma 300, KEITLEY 2400 picoammeter/voltage source, Shimadzu UV-1800 spectrophotometer, and Agilent Technologies Cary Eclipse fluorescence spectrophotometer were used for transmission electron microscopy (TEM), zeta potential measurements, X-ray diffractometry (XRD), Fourier-transform infrared spectroscopy (FTIR), X-ray photoelectron spectroscopy (XPS), micro-Raman spectroscopy, PVD thermal evaporation, scanning electron microscopy (SEM), *I*–*V*/*C*–*V* measurements, UV–vis spectroscopy, and steady-state fluorescence spectroscopy, respectively.

### CDs synthesis

2.5 g of the powdered *Rheum ribes* plant was placed in an autoclave bottle, and 50 mL of pure water was added to the bottle. This aqueous solution was placed in an autoclave and hydrothermal synthesis was carried out at 125 °C for 12 h. After the reaction was completed, the aqueous suspension was brought to room temperature and filtered with ordinary filter paper. The filtrate was passed through a 0.22 µm membrane filter and centrifuged at 10000 rpm for 30 min. The supernatant was removed by decantation and a stock solution was prepared to be used in the studies. The prepared stock solution was stored at 4 °C to prevent contamination [15].

### Schottky diode fabrication

**Figure 1:**
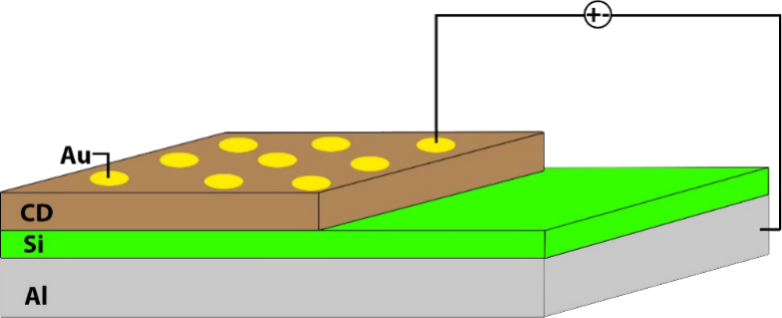
Schematic representation of the Schottky diode based on CDs.

An n-type silicon substrate was used for Schottky diode fabrication. In the first step, the silicon substrate was cut to approximately 1 cm^2^ in size and cleaned by washing it in acetone, methanol, and pure water for 10 min each. An ohmic contact was made with aluminum on the cleaned sample at 1 × 10^−7^ Torr vacuum in a PVD thermal evaporation device. Approximately 30 μL of the CDs stock solution was taken and diluted with 5 mL of pure water; 100 μL of this solution was dripped onto the silicon substrate and evaporated at room temperature. In the last stage, the diode structure was obtained by evaporating gold onto the created CDs film using the shadow mask technique (Figure 1). The size of the diode and the thickness of coated CDs film were 1 cm^2^ and ca. 566 nm, respectively.

## Results and Discussion

### Structural and optical characterization of synthesized CDs

**Figure 2:**
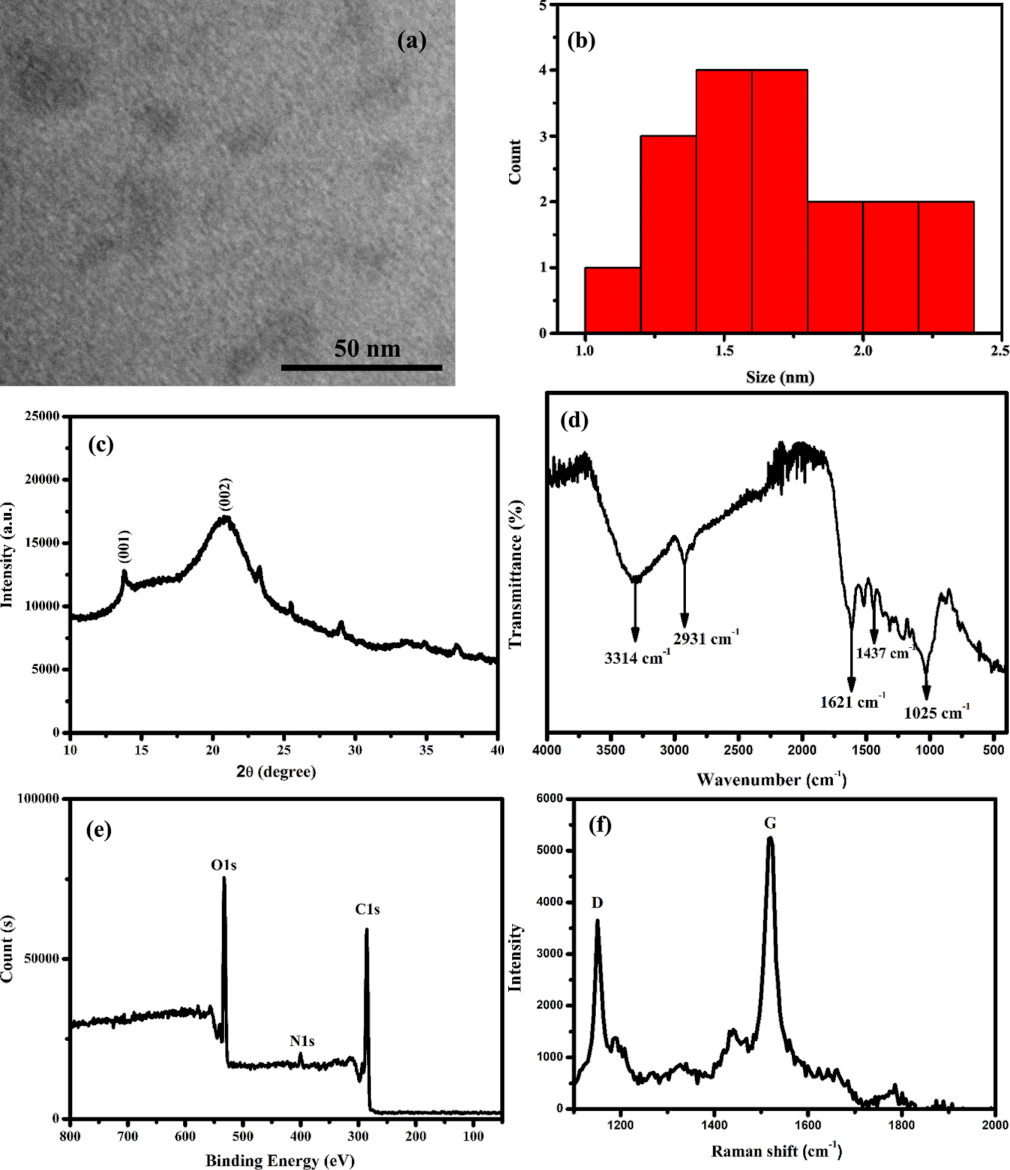
(a) TEM image, (b) size distribution, (c) XRD pattern, (d) FTIR spectrum, (e) XPS spectrum, and (f) Raman spectrum of the CDs.

The morphological and chemical structures of the CDs obtained from *Rheum ribes* were determined by TEM, FTIR, XRD, and XPS analyses. TEM shows that the CDs have a monodisperse distribution and a spherical structure (Figure 2a) [16]. Additionally, ImageJ software was used to analyze the size distribution of CDs. Most of the CDs were in the range of 1.0–2.5 nm with an average size of 1.5 nm (Figure 2b). The XRD pattern of the prepared CDs is shown in Figure 2c. The weak and broad peak observed at 2θ = 21° is due to the weakly crystalline structure of the synthesized CDs and the presence of graphitic carbon, indicating that it is amorphous in nature [17]. FTIR measurements were taken to determine the functional groups on the surface of the CDs. As seen in Figure 2d, peaks associated with stretching vibrations of O–H, C–H, carbonyl (C=O), aromatic C=N, and C–O–C groups were observed at 3314, 2931, 1621, 1437, and 1025 cm^−1^, respectively. These functional groups confer watersolubility to the CDs [11,18]. To corroborate the FTIR measurements, elemental characterization of CDs was carried out by XPS. As seen in Figure 2e, three peaks were observed at 533, 399, and 285 eV, corresponding to O 1s, N 1s, and C 1s, respectively. In addition, the elements fractions in the CDs were determined as 73.3 atom % C, 24.0 atom % O and 2.7% atom N. The FTIR and XPS results are in agreement.

Raman spectroscopy was also carried out to confirm the presence of aromatic carbon atoms in the newly synthesized CDs. The D band was observed at 1150 cm^−1^, and the G band was observed at 1519 cm^−1^ (Figure 2f). The D band is associated with defects in the graphite lattice, while the G band is attributed to the vibrations of sp^2^-bonded carbon atoms in a 2D hexagonal lattice. The degree of defects in the CDs can be calculated from the relative intensity ratio (*I*_D_/*I*_G_) of two peaks of D band and G band. *I*_D_/*I*_G_ was calculated to be approximately 0.69, indicating that the prepared CDs exhibited comparable amounts of the two carbon species [19].

In addition, the surface charge of the CDs was found to be −5.77 mV by zeta potential measurements. This value shows that there are more negatively charged carboxyl and hydroxy groups on the surface of the CDs than positively charged amino groups [20].

**Figure 3:**
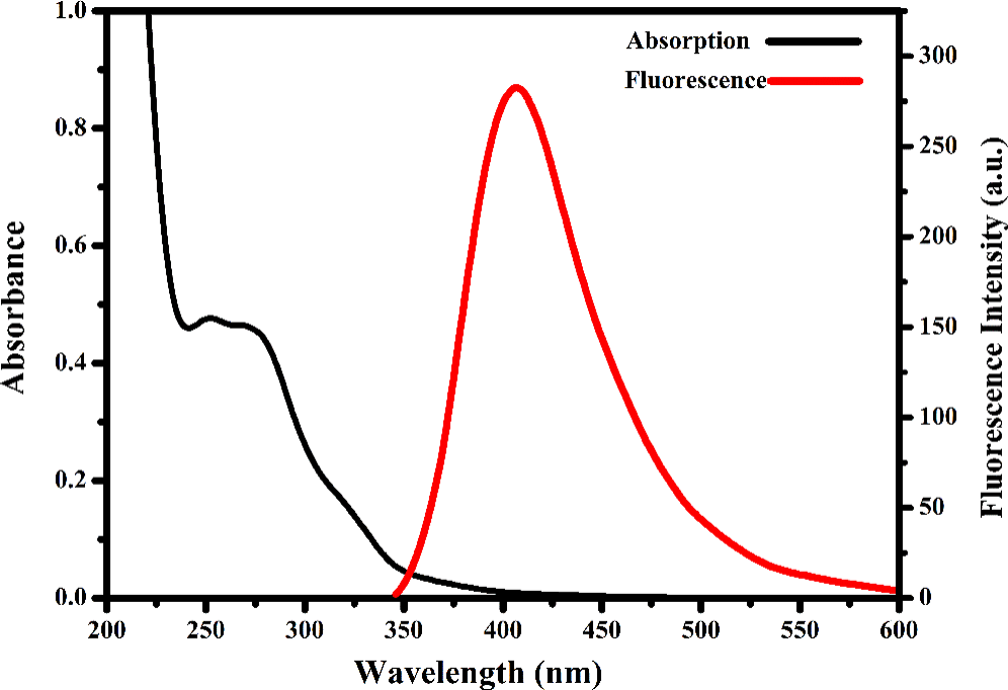
UV–vis absorption and fluorescence spectra of the CDs (λ_exc_ = 320 nm).

Absorption and fluorescence measurements were taken for optical characterization of the CDs. The absorption peaks observed at 229 and 251 nm arise from the π–π* transitions of the conjugated electrons and the n–π* transitions of the oxygen and nitrogen heteroatoms in the structure, respectively [21]. When the excitation wavelength was 320 nm, the CDs exhibited a strong fluorescence peak at approximately 394 nm (Figure 3).

**Figure 4:**
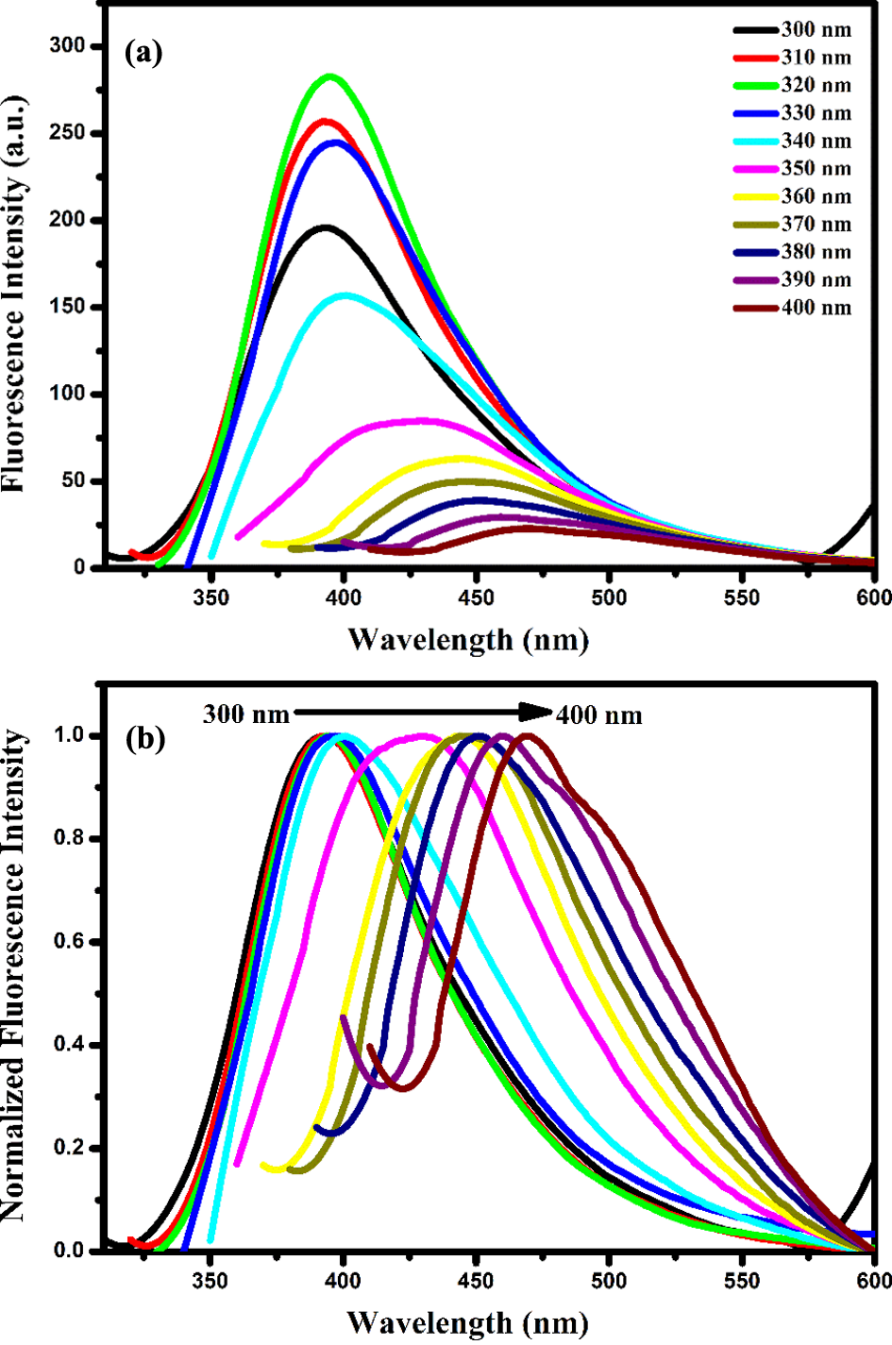
(a) Fluorescence and (b) normalized fluorescence spectra of the CDs at various excitation wavelengths in the range from 300 to 400 nm.

The dependence of the fluorescence intensity of the CDs on the excitation wavelength was also studied. Fluorescence measurements were performed at different excitation wavelengths in the range of 300–400 nm in 10 nm intervals (Figure 4a). As the excitation wavelength increased, the fluorescence intensity of the CDs decreased, and the fluorescence peak maximum shifted to red (Figure 4b). It has been stated that this change in the fluorescence of the CDs is caused by particles of different sizes in the structures or emission traps on the surface [22]. Moreover, the fluorescence quantum yield of the synthesized CDs was calculated by the Parker–Rees equation:


[1] $$\Phi_{\text{s}}=\Phi_{\text{r}}\left(\frac{D_{\text{s}}}{D_{\text{r}}}\right)\left(\frac{n_{\text{s}}^{2}}{n_{\text{r}}^{2}}\right)\left(\frac{1-10^{-{\text{OD}}_{\text{r}}}}{1-10^{-{\text{OD}}_{\text{s}}}}\right),$$

where *D* is the integrated area under the corrected fluorescence spectrum, *n* is the refractive index of the solvent, and OD is the optical density at the excitation wavelength (λ_exc_ = 320 nm). The subscripts “s” and “r” refer to the sample and reference solutions, respectively. Quinine sulfate (Φ_f_ = 0.55 in 0.5 M H_2_SO_4_) was used as the reference compound [15]. According to this equation, the quantum yield of the CDs was calculated as 0.03.

### Schottky diode fabrication

**Figure 5:**
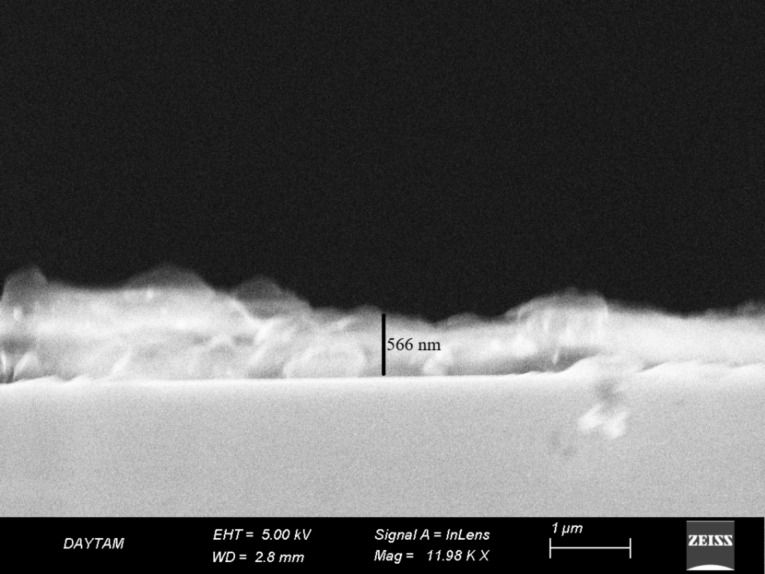
SEM image of Si/CDs/Au-based Schottky diode.

The usability of these newly synthesized CDs in an application area was also discussed. For this purpose, a metal (Au)–semiconductor (CDs) junction-based thin film device was produced. SEM images were taken to determine the structure of the coated CDs film (Figure 5); the CDs film thickness was determined as ca. 566 nm.

**Figure 6:**
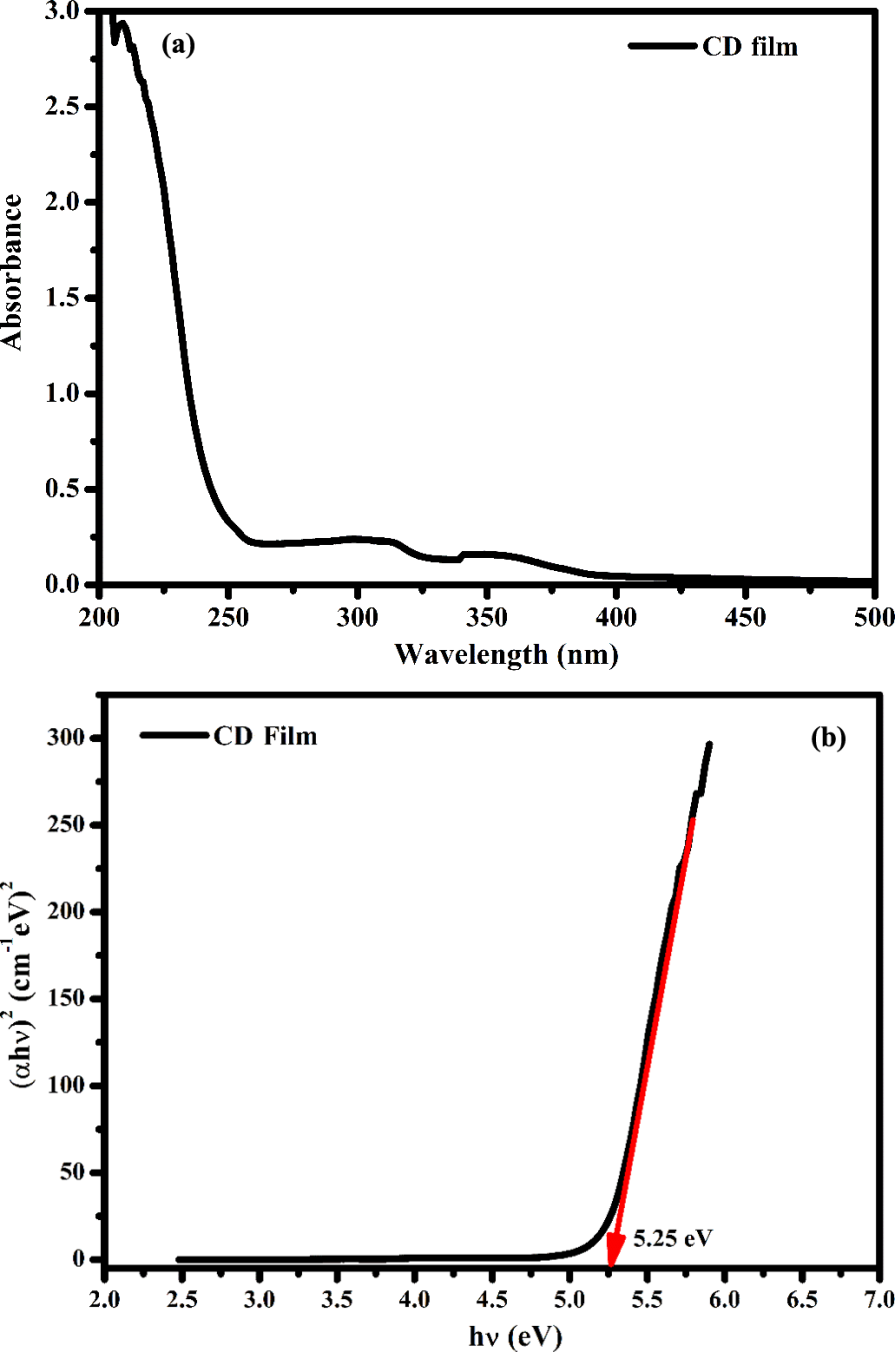
(a) UV–vis absorption spectra and (b) bandgap of the CDs layer.

In addition, a UV–vis absorption spectrum of the CDs layer was taken (Figure 6a), and the bandgap value of the layer was determined from the graph of *h*ν versus (α*h*ν)^2^ using the Tauc equation [23]. The bandgap value of the CDs layer was 5.25 eV (Figure 6b).

**Figure 7:**
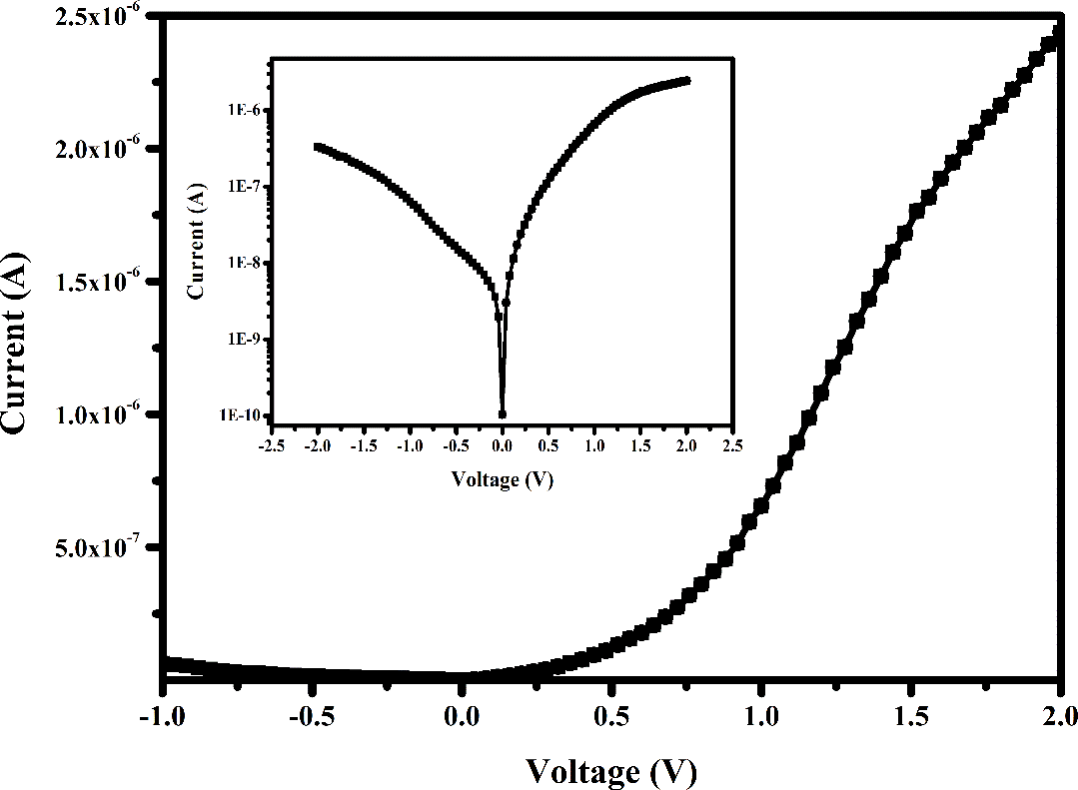
*I*–*V* characteristics of the Si/CDs/Au-based Schottky diode.

The current–voltage (*I*–*V*) characteristics of the Si/CDs/Au-based Schottky diode were investigated. *I*–*V* measurements of the CDs-based thin film device were carried out using a semiconductor parameter analyzer between −2.5 V and +2.5 V at room temperature (Figure 7). The interface between Au and CDs exhibited a nonlinear rectification behavior, indicating the formation of a Schottky diode [24].

The electrical properties of the Si/CDs/Au diode were determined using standard thermionic emission theory [25]. According to this theory,


[2] $$I=I_{0}\exp\left[\frac{q\left(V-IR_{\text{s}}\right)}{nk_{\text{B}}T}\right],$$

where *n*, *IR*_s_, *V*, *q*, *k*_B_, *T*, and *I*_0_ are the ideality factor, the voltage drop on the series resistance (*R*_s_), the applied bias voltage, the electronic charge, Boltzmann’s constant, the temperature in Kelvin, and the reverse saturation current, respectively. The reverse saturation current of the Schottky diode can be written as:


[3] $$I_{0}=AA^{*}T^{2}\exp\left(\frac{-q\Phi_{\text{b}}}{k_{\text{B}}T}\right),$$

where Φ_b_ is the effective barrier height, *A* is the device area, and A* is the Richardson constant (112 A·cm^−2^·K^−2^ for n-type Si [26]). Φ_b_ can be obtained from Equation 3. The value of *n* is calculated from the slope of semi-logarithmic *I*–*V* plots and is given as


[4] $$n=\frac{q}{k_{\text{B}}T}\left(\frac{\text{d}V}{\text{d}\left(\ln I\right)}\right).$$

The ideality factor and the value of the measured barrier height Φ_b_ for the synthesized CDs were 9.1 and ca. 0.364 eV, respectively. Considering the CDs-based Schottky diode, it can be said that although its rectification property is good, it exhibits a non-ideal diode behavior with a high ideality factor [27].

## Conclusion

In this study, *Rheum ribes*, which grows wild in the Eastern Anatolia region of Turkey, was used as a carbon source for the first time, and fluorescent carbon dot structures (CDs) were successfully synthesized in a single step by the hydrothermal synthesis method. Morphology, size, and surface charge of these new synthesized CDs were determined, and their structural and optical characterization was performed. The usability of the new CDs in diode fabrication as an application area was discussed. The layer thickness and bandgap energy for the prepared CDs film from SEM and UV–vis absorption measurements were 566 nm and 5.25 eV, respectively. From *I*–*V* measurements of the diode at room temperature, it was determined that the designed device exhibited a non-linear rectification behavior, which was in agreement with literature. It was found that although the rectification of the CDs-based diode is good, it exhibits a non-ideal diode behavior with a high ideality factor. This study, in which CDs obtained from a completely natural product were used as an interface, showed that it may be possible to obtain CDs-based electronic devices for advanced technology in the future.

## Data Availability

The data that supports the findings of this study is available from the corresponding author upon reasonable request.
